# Nrdp1S, short variant of Nrdp1, inhibits human glioma progression by increasing Nrdp1‐mediated ErbB3 ubiquitination and degradation

**DOI:** 10.1111/jcmm.12735

**Published:** 2015-11-27

**Authors:** Yuxuan Wu, Lei Wang, Hanmo Bao, Shenshan Zou, Chunling Fu, Hui Gong, Yong Gao, Yuan Tang, Rutong Yu, Hengliang Shi

**Affiliations:** ^1^ Insititute of Nervous System DiseasesXuzhou Medical CollegeXuzhouChina; ^2^ The School of Clinical MedicineXuzhou Medical CollegeXuzhouChina; ^3^ The Graduate SchoolXuzhou Medical CollegeXuzhouChina; ^4^ Brain HospitalAffiliated Hospital of Xuzhou Medical CollegeXuzhouChina; ^5^ Institute of Blood DiseasesXuzhou Medical CollegeXuzhouChina

**Keywords:** Nrdp1, Nrdp1S, ubiquitination, ErbB3, glioma

## Abstract

The ubiquitin ligase neuregulin receptor degradation protein 1 (Nrdp1) is involved in the induction of apoptosis and suppression of tumour formation. We previously showed that it was expressed at lower levels in human glioma tissues compared with normal brain tissues. However, the mechanism underlying this is unclear. Here, we reported that a novel short variant (Nrdp1S), lacking 71 amino acids at the N‐terminal, was expressed in normal human brain tissue, but absent from glioma tissues. Similar to Nrdp1, Nrdp1S could be degraded by the proteasomal pathway, but exhibited an even longer half‐life than Nrdp1. Nrdp1S was also shown to form a heterodimer with Nrdp1, which increased its stability, thereby augmenting the Nrdp1‐mediated ubiquitination and degradation of ErbB3. EdU incorporation, MTT assay and *in vitro* colony formation demonstrated that Nrdp1S significantly inhibited the cell tumourigenicity. These results together suggest that Nrdp1S is a tumour suppressor that which potentiates the Nrdp1‐mediated ubiquitination and degradation of ErbB3. An Nrdp1S deficiency may also be an important factor in the loss of Nrdp1.

## Introduction

Malignant glioma is the most common form of brain cancer. Affected patients are usually treated with a combined approach of surgery, chemotherapy and radiation therapy 1, 2, 3, 4, but the median survival time is only 12–15 months. Therefore, an understanding of the mechanisms underlying disease pathogenesis is critical to identify specific molecular targets that could be used as a treatment for glioma.

Neuregulin receptor degradation protein 1 (Nrdp1), a member of the RING/B‐box/coiled‐coil or tripartite interaction motif (TRIM) family of proteins, is a ubiquitin ligase that reported to mediate the ubiquitination of several proteins including ErbB3, several type1 cytokine receptors, the Toll‐like receptor signalling adaptor protein myeloid differentiation primary response gene 88 (Myd88), the BIR repeat containing ubiquitin‐conjugating enzyme, the CCAAT/enhancer‐binding protein β and the ubiquitin ligase Parkin.

Structural analysis has shown that Nrdp1 contains four domains typical of RING family proteins 5. Amino acids (aa) 1–134 make up the RING finger domain and B‐box domain, while aa 135–317 form the coiled‐coil domain and substrate‐binding region. Nrdp1S, a short form of Nrdp1 that lacks the N‐terminal 71 aa, is deficient in the RING finger domain, which is involved in self‐ubiquitination. However, the role of Nrdp1S has not yet been elucidated.

The ErbB family of tyrosine kinase receptors, also known as Human epidermal receptors (HER) in humans, comprises four members: erbB1/Epidermal growth factor receptor (EGFR), erbB2/neu, erbB3 and erbB4 6, 7, 8. Unlike most other membrane receptors, ErbB3 does not undergo degradation by lysosomes 9, 10, but is degraded by proteasomes catalysed by Nrdp1 5, 11. The overexpression of ErbB3 has been reported to contribute to tumour malignancy and therapeutic resistance in cancers 12, 13, 14, 15. Furthermore, the loss of Nrdp1 enhances ErbB2/ErbB3‐dependent breast cancer cell growth 16. However, it is not clear whether Nrdp1S is associated with the loss of Nrdp1 and the dysregulation of ErbB3.

In this study, we investigated the expression of Nrdp1S in human brain and glioma tissues, and studied its interaction with Nrdp1 in the role of a tumour suppressor.

## Materials and methods

### Antibodies

Nrdp1 antibody (A310‐012A) was purchased from Bethyl Laboratories (Montgomery, TX, USA). FLAG (F3165) antibody was from Sigma‐Aldrich (St. Louis, MO, USA). Green fluorescent protein (GFP) (sc‐8334) antibody was from Santa Cruz (Santa Cruz, CA, USA). ErbB3 antibody (05‐390) was from Millipore (Billerica, MA, USA). Antibodies specific for Hemagglutinin tag (HA) (#3724S) and β‐actin (#4970S) were purchased from Cell Signaling Technology (Danvers, MA, USA).

### Tissue samples

Six specimens of human non‐tumourous brain tissues (internal decompression in cerebral trauma) and eight specimens of glioma tissues (surgical resection) were collected at the Affiliated Hospital of Xuzhou Medical College (Xuzhou, China). Surgically removed tissues were sampled for histological diagnosis and the remaining tissues were immediately frozen in liquid nitrogen and stored at −80°C in the fridge. The informed consent was obtained from all patients. The methods were carried out in accordance with the approved guidelines of Xuzhou Medical College Research Ethics Committee. All experimental protocols were approved by Xuzhou Medical College Licensing Committee.

### Constructs and production of lentivirus

For overexpression of Nrdp1 or Nrdp1S with lentivirus, the Nrdp1 or Nrdp1S cDNA was inserted into the pWPXLd‐puro plasmid using *BamH* I and *Mlu* I sites, which express GFP‐fused Nrdp1 or Nrdp1S. The viruses were propagated in HEK293T cells by cotransfecting the recombinant plasmids with the helper plasmids. Cell transfection was performed with Polyjet (SignaGen laboratories, Rockville, MD, USA) as described in the manufacturer's protocol.

### Establishment of stable cell lines

The establishment of stable cell lines was performed as previously described 17. For overexpression of Nrdp1 or Nrdp1S in human glioma cell U251, the U251 cells were infected by GFP, GFP‐Nrdp1 or GFP‐Nrdp1S viruses respectively. Forty‐eight hours after infection, the cells were continuously cultured in the medium containing 2.5 μg/ml puromycin. The surviving cells were expanded cultured to cell lines stably expressing GFP, GFP‐Nrdp1 and GFP‐Nrdp1S.

### Transient transfection of ErbB3 siRNAs

Transfection of ErbB3 siRNAs oligo was performed with the Lipofectamine^™^ 2000 transfection reagent (Invitrogen, Carlsbad, CA, USA) following the manufacturer's instructions. ErbB3 siRNA and control siRNA duplexes purchased from GenePharma (Shanghai, China) were listed as follows: ErbB3 siRNA, 5′‐GGCCAUGAAUGAAUUCUCUACUCUA‐3′; control siRNA‐NC, 5′‐UUCUCCGAACGUGUCACGUAUCAGC‐3′.

### RNA extraction, cDNA synthesis and quantitive RT‐PCR

RNA was extracted from frozen specimens (six specimens of human non‐tumourous brain tissues and eight specimens of glioma tissues) and the cDNA was synthesized using reverse transcription reagents (Roche, Basel, Switzerland) according to the manufacturer's protocol. Quantitive RT‐PCR was performed on an ABI 7300 real‐time PCR instrument (Applied Biosystems, Carlsbad, CA, USA) using SYBR Green. Primers for the amplification of Nrdp1S and β‐actin are as follows: Nrdp1S‐F, ACCAGCTTTTGATACAGCCA; Nrdp1S‐R, TGGCTGTATCAAAAGCTGGT; β‐actin‐F, CATGTACGTTGCTATCCAGGC; β‐actin‐R, CGCTCGGTGAGGATCTTCATG. The products were 143 and 195 bp respectively.

### Western blotting

At the designated time, the cells were lysed and equal amount of protein lysates were subjected to 10% SDS‐PAGE and then transferred to 0.45 μm pore size PVDF membrane (Millipore). After blocking with 5% non‐fat milk, the membrane was probed with primary antibodies (Nrdp1, ErbB3, GFP, FLAG, HA and β‐actin) at 4°C overnight and secondary antibodies at room temperature for 1 hr. Bound antibodies were detected by the Pierce ECL Plus Western Blotting Substrate (Thermo Fisher, Waltham, MA, USA) and exposed to X‐ray films. Band densities were quantified by Image J Software (Wayne Rasband, National Institutes of Health, MD). The relative amount of proteins was determined by normalizing the densitometry value of interest to that of the loading control.

### Immunoprecipitation

HEK293T cells were transiently transfected with the indicated plasmids. Twenty‐four hours after transfection, the cells were lysed in a Triton‐X‐100‐based lysis buffer (1% Triton‐X‐100, 150 mM NaCl, 20 mM 4‐(2‐Hydroxyethyl)‐1‐piperazineethanesulfonic acid (HEPES), pH 7.4, 2 mM ethylenediaminetetraacetic acid, 5 mM MgCl_2_) supplemented with protease inhibitor for 20 min. on ice. The nuclear and cellular debris were cleared by centrifugation. Of total protein, 1 mg was subjected for immunoprecipitation with the indicated antibody. The immunoprecipitates were washed five times in lysis buffer, and proteins were recovered by boiling the beads in SDS sample buffer and analysed with Western blotting.

### EdU incorporation assay

Cells were seeded into 96‐well plates at 7 × 10^3^ cells per well. Twenty‐four hours later, the cells were exposed to 50 μM of 5‐ethynyl‐20‐deoxyuridine (EdU; Ribobio, Guangzhou, China) for additional 2 hrs at 37°C. Then, the cells were fixed with 4% paraformaldehyde for 20 min. and treated with 0.5% Triton‐X‐100 for another 20 min. at room temperature. After being washed with PBS for five times, the cells were reacted with 100 μl of 1× Apollo^®^ reaction cocktail for 30 min. Thereafter, the DNA contents of cells were stained with 100 μl of Hoechst 33342 (5 μg/ml) for 20 min. and visualized under a fluorescent microscope (IX71; Olympus, Tokyo, Japan).

### MTT assay

Three thousand cells in 200 μl of medium were plated in 96‐well plates and grown under normal conditions. The 3‐(4, 5‐dimethylthiazol‐2‐yl)‐2, 5‐diphenyltetrazolium bromide (MTT; Sigma‐Aldrich) was added into the medium to the final concentration of 0.5 mg/ml when the cells adherent to the plate. Following incubation for 4 hrs, 150 μl of Dimethyl sulfoxide (DMSO) was replaced to dissolve the crystals. Viable cells were counted every day by reading the absorbance at 490 nm using SynergyMx Multi‐Mode Microplate Reader (Biotek, Winooski, VT, USA).

### Plate colony formation

Two hundred cells were seeded in 6 cm dish and cultured for 10 days. The cells were fixed with methanol and stained with 0.05% crystal violet to assess colony staining. After washing with PBS, the plates were photographed with a camera. Colonies containing more than 50 cells were counted.

### Statistical analysis

The software of SPSS package version 13.0 (SPSS Inc, Chicago, IL) was used to perform statistical analyses. Data are presented as the mean ± S.E.M. Statistical significance was determined using Student's *t*‐test, with *P* < 0.05 considered significant.

## Results

### Nrdp1S is expressed in normal brain tissues but is absent from glioma tissues

**Figure 1:**
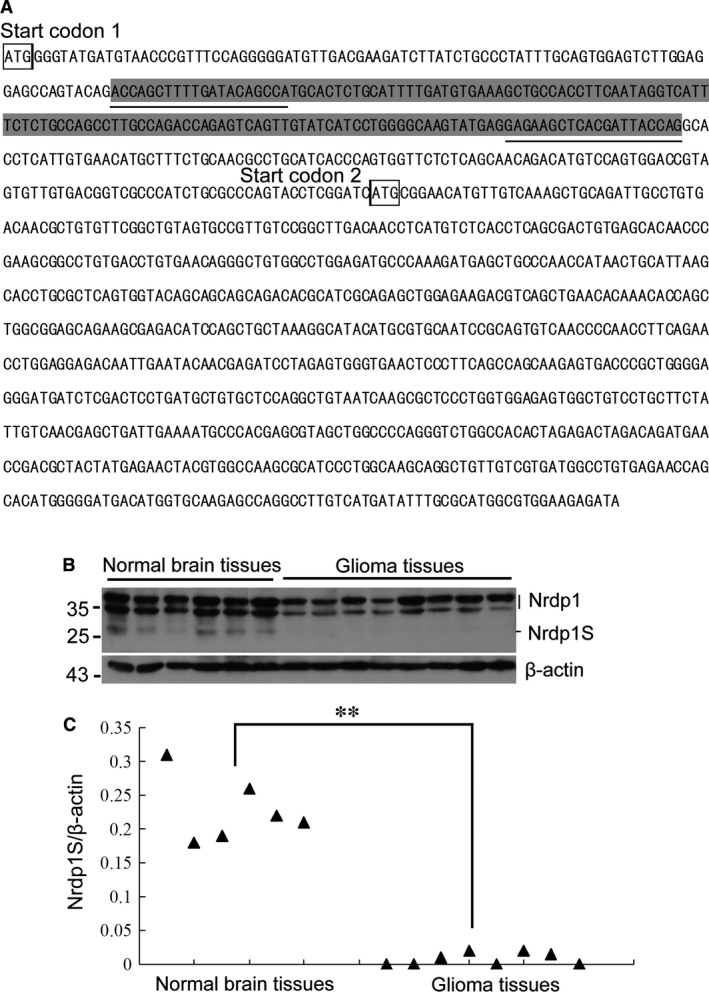
Nrdp1S is expressed in normal brain tissues, but is absent from glioma tissues. (**A**) Schematic showing the 143 bp insertion (marked by a shaded background) in the 5′ region, which results in the use of a downstream start codon (start codon 2) in the transcription of Nrdp1S protein, which lacks the N‐terminal 71 amino acids. The underlined sequence represents the location of primer binding. (**B**) Western blotting showing the expression of Nrdp1S in normal human brain and glioma tissues. (**C**) Quantitive RT‐PCR showing the expression of Nrdp1S mRNA in normal human brain and glioma tissues,**P < 0.01.

GenBank contains four transcript variants of *Nrdp1*. Variants 1, 3 and 4 encode full‐length Nrdp1 proteins that use start codon 1 for transcription. However, variant 2 includes an additional 143 bp insertion in the 5′ region, which results in the use of a downstream start codon (start codon 2) encoding an isoform with a shorter N‐terminus lacking 71 aa, defined as Nrdp1S (Fig. 1A).

To detect Nrdp1S expression, total protein was isolated from normal human brain tissues and glioma tissues and subjected to Western blotting using an anti‐Nrdp1 antibody. As shown in Figure 1B, the top two bands were Nrdp1 (35 and 37 kD), likely representing phosphorylated and unphosphorylated forms, while the band of lower molecular weight represents Nrdp1S (27 kD). Interestingly, the Nrdp1S band was presented in normal brain tissues, but absent from glioma tissues. Nrdp1S mRNA expression was also confirmed by quantitative RT‐PCR using the primers Nrdp1S‐F and Nrdp1S‐R, which are Nrdp1S‐specific. Nrdp1S mRNA was observed in normal brain tissue, but was barely detectable in glioma tissue (Fig. 1C), which was consistent with the Western blotting results.

### Nrdp1S is degraded by the proteasome pathway, similarly to Nrdp1, but undergoes less auto‐ubiquitination

**Figure 2:**
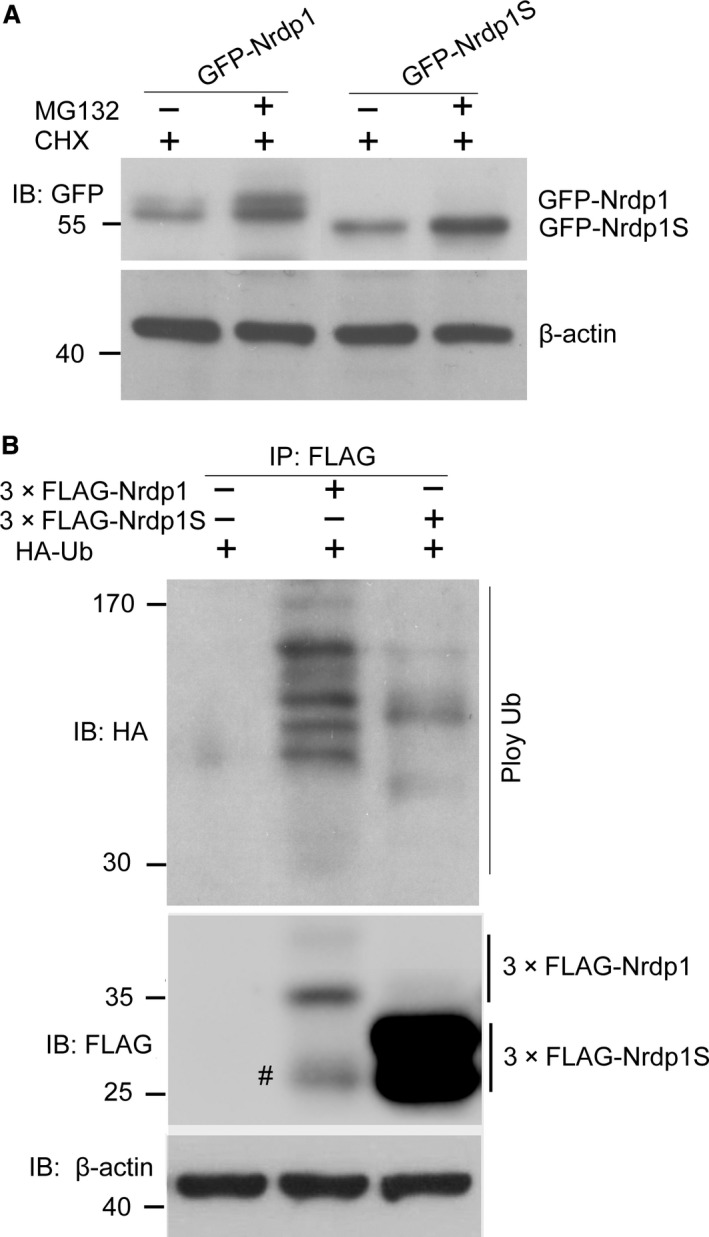
Nrdp1S can be degraded by the proteasomal pathway, similar to Nrdp1, but undergoes less auto‐ubiquitination than Nrdp1. (**A**) Western blotting showing that degradation of Nrdp1 and Nrdp1S can be blocked by MG132. (**B**) Ubiquitination assay showing that Nrdp1S undergoes less ubiquitination compared with Nrdp1, ^#^unspecific.

Nrdp1 is involved in the ubiquitin‐mediated degradation of several target proteins, including Nrdp1 itself 18, 19. To confirm whether Nrdp1S is also subjected to similar degradation through the ubiquitn–proteasome pathway, we developed U251 cell lines overexpressing GFP‐Nrdp1 and GFP‐Nrdp1S by lentiviral infection. Cells were cultured in the presence or absence of MG132 for 6 hrs, then proteins were extracted and analysed by Western blotting. As shown in Figure 2A, the degradation of both Nrdp1 and Nrdp1S was blocked in the presence of MG132, suggesting that the degradation pathways are proteasome‐dependent.

Some E3 ligases possessing RING domains, such as Mdm2, XIAP and c‐IAP1 20, 21, 22, are auto‐ubiquitinated in a RING domain‐dependent manner. To determine whether ubiquitination is involved in the Nrdp1S degradation pathway, we transiently cotransfected 3× FLAG‐Nrdp1 or 3× FLAG‐Nrdp1S with HA‐Ub into HEK293T cells. Cell lysates were immunoprecipitated with an anti‐FLAG antibody, and the immunoprecipitates were detected using HA and FLAG antibodies. As shown in Figure 2B, both Nrdp1 and Nrdp1S underwent ubiquitination, but the ubiquitination of Nrdp1S was at a lower level compared with Nrdp1. Taken together, we can conclude that both Nrdp1 and Nrdp1S can be ubiquitinated and subsequently degraded by proteasomes.

### Nrdp1S promotes the Nrdp1 mediated ubiquitination and degradation of ErbB3

**Figure 3:**
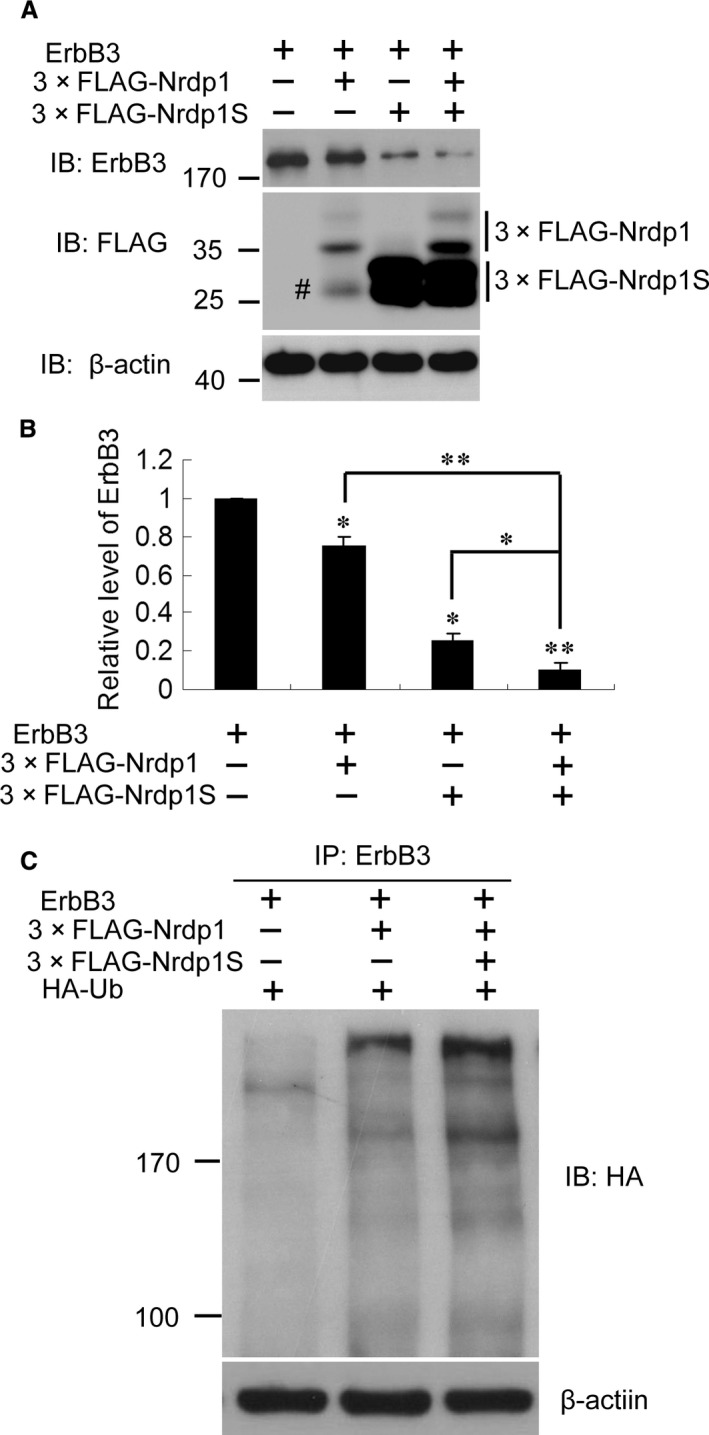
Nrdp1S promotes the Nrdp1‐mediated ubiquitination and degradation of ErbB3. (**A**) Representative Western blots showing that Nrdp1S promotes the degradation of ErbB3. (**B**) Histogram showing the promotion of ErbB3 degradation by Nrdp1S. (**C**) Representative Western blots showing that ErbB3 ubiquitination is augmented by the co‐overexpression of Nrdp1S. ^#^unspecific. *P < 0.05; **P < 0.01.

It was previously reported that the overexpression of wild‐type Nrdp1 suppresses ErbB3 levels by promoting its ubiquitination and degradation 5, 12. To explore the role of Nrdp1S in regulating ErbB3, different cotransfection experiments were set up in HEK293T cells. As expected, the overexpression of Nrdp1 suppressed ErbB3 levels in transfected cells. Interestingly, ErbB3 levels were further decreased by the co‐overexpression of Nrdp1S (Fig. 3A and B). Accordingly, ErbB3 ubiquitination was augmented by the co‐overexpression of Nrdp1S (Fig. 3C). These results indicate that Nrdp1S promotes the Nrdp1 mediated ubiquitination and degradation of ErbB3.

### Nrdp1S increases the stability of Nrdp1 by forming an Nrdp1–Nrdp1S heterodimer

**Figure 4:**
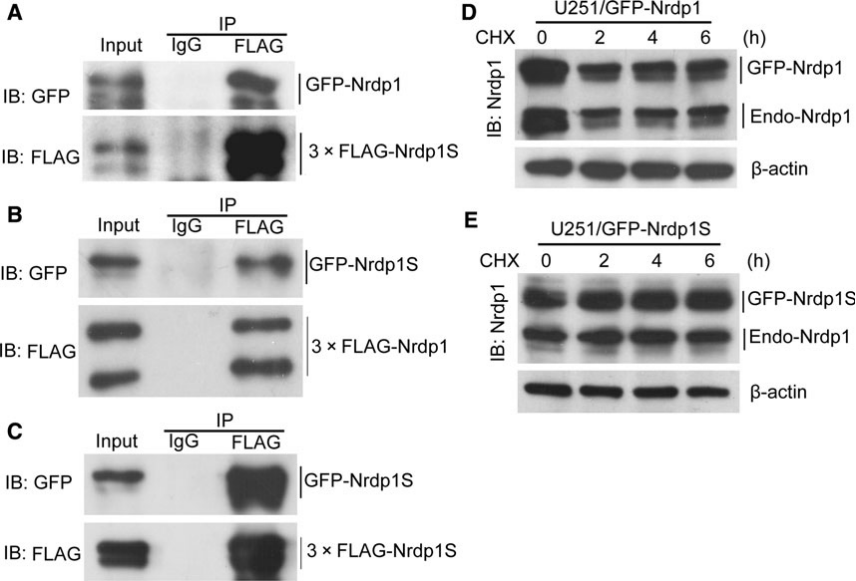
Nrdp1S increases the stability of Nrdp1 by assembling into an Nrdp1–Nrdp1S heterodimer. (**A**) Co‐immunoprecipitation assay demonstrating that Nrdp1 forms homodimers with itself. (**B**) Co‐immunoprecipitation assay revealing that Nrdp1 can also interact with Nrdp1S to form a heterodimer. (**C**) Co‐immunoprecipitation assay showing that Nrdp1S forms homodimers with itself. (**D**) Western blotting showing the stability of exogenous GFP‐Nrdp1 and endogenous Nrdp1 in GFP‐Nrdp1 overexpressing cells. (**E**) Western blotting showing the stability of exogenous GFP‐Nrdp1S and endogenous Nrdp1 in GFP‐Nrdp1S overexpressing cells.

Nrdp1 has potent auto‐ubiquitination activity, which leads to its constitutive degradation and short half‐life in transformed cells 11, 17. Nrdp1 auto‐ubiquitination was also shown to be dependent on the formation of Nrdp1–Nrdp1 homodimers involving the coiled‐coil domains of the proteins 19. It is also showed that the auto‐ubiquitination of Nrdp1 dependents on the formation of Nrdp1*Nrdp1 homodimer by their coiled‐coil domain. Our earlier results (Fig. 3) led us to speculate that Nrdp1S may play a role in the regulation of Nrdp1 stability. To address this, we first verified the formation of Nrdp1–Nrdp1 homodimers by cotransfecting HEK293T cells with GFP‐tagged Nrdp1 and 3× FLAG‐tagged Nrdp1. Immunoprecipitation with an anti‐FLAG antibody revealed that GFP‐Nrdp1 associates with 3× FLAG‐Nrdp1 (Fig. 4A), which is consistent with previous report 19.

We next determined whether Nrdp1S could also interact with Nrdp1 to form a heterodimer by cotransfecting HEK293T cells with 3× FLAG‐tagged Nrdp1 and GFP‐tagged Nrdp1S. As shown in Figure 4B, GFP‐Nrdp1S could be immunoprecipitated by 3× FLAG‐Nrdp1, demonstrating that Nrdp1 may indeed physically interact with Nrdp1S in mammalian cells. Finally, to examine whether Nrdp1S is able to form a homodimer, cell lysates from HEK293T cells expressing GFP‐Nrdp1S and 3× FLAG‐Nrdp1S were subjected to co‐immunoprecipitation. As shown in Figure 4C, GFP‐Nrdp1S was able to co‐precipitate 3× FLAG‐Nrdp1S. Together, these results suggest that both Nrdp1 and Nrdp1S can interact with themselves to form homodimers or with each other to form an Nrdp1–Nrdp1S heterodimer.

Given the Nrdp1S–Nrdp1 interaction, we investigated whether Nrdp1S could regulate the stability of Nrdp1 by treating 251/GFP‐Nrdp1 and 251/GFP‐Nrdp1S cells with 25 μg/ml CHX. As shown in Figure 4D, both exogenous GFP‐Nrdp1 and endogenous Nrdp1 rapidly degraded after 2 hrs treatment with CHX in GFP‐Nrdp1 overexpressing cells; however, exogenous GFP‐Nrdp1S and endogenous Nrdp1 levels were almost unchanged in GFP‐Nrdp1S overexpressing cells (Fig. 4E). This indicates that Nrdp1S has a longer half‐life than Nrdp1, and that overexpressing Nrdp1S increases the stability of endogenous Nrdp1. Taken together, these results suggest that Nrdp1S may function as a modulator of Nrdp1 auto‐ubiquitination and degradation, and exhibit a synergistic role in controlling the level of ErbB3 substrate.

### Nrdp1S potentiates the Nrdp1‐mediated inhibition of human glioma cell proliferation

**Figure 5:**
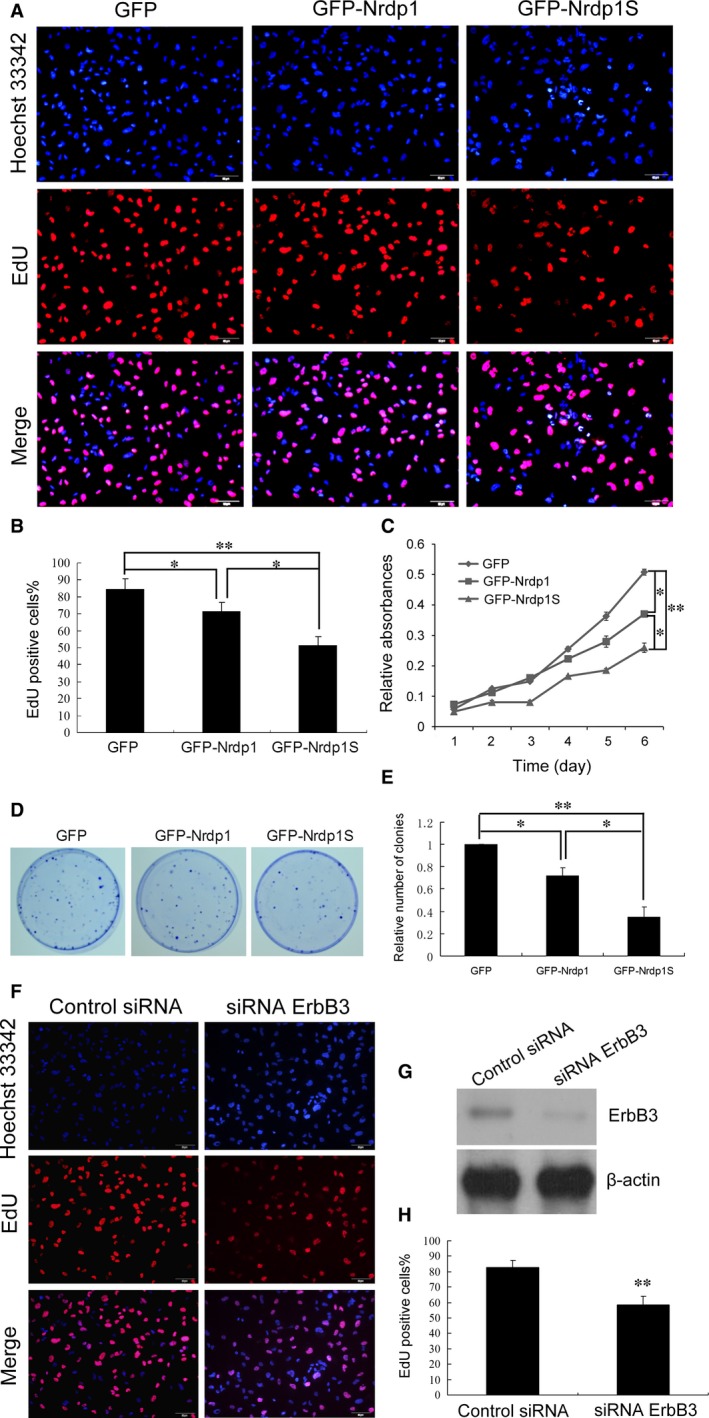
Nrdp1S potentiating the Nrdp1‐mediated inhibition of human glioma cell proliferation. (**A** and **B**) EdU assay showing that Nrdp1S augments the Nrdp1‐mediated inhibition of proliferation in U251 cells; scale bar, 50 μm. (**C**) MTT assay showing that Nrdp1S augments the Nrdp1‐mediated inhibition of proliferation in U251 cells. (**D** and **E**) Colony formation assay showing that Nrdp1S augments the Nrdp1‐mediated inhibition of proliferation in U251 cells. (**F** and **G**) EdU assay showing that ErbB3 knockdown inhibits the proliferation of U251 cells. (H) Histogram showing that ErbB3 knockdown inhibits the proliferation of U251 cells. Data are expressed as the mean ± S.E.M. from three independent experiments. *P < 0.05; **P < 0.01.

Nrdp1 has been reported to be a tumour suppressor through reducing the level of ErbB3 and restricting the progression of cancers 11, 16. To determine whether the inhibitory effect of Nrdp1 on glioma progression could be potentiated by Nrdp1S, we subjected U251 cell lines overexpressing GFP, GFP‐Nrdp1 and GFP‐Nrdp1S to MTT and EdU assays. Nrdp1 overexpression was found to inhibit the proliferation of U251 cells, which was augmented by Nrdp1S (Fig. 5A–C). Additionally, the cell colony formation experiment further demonstrated that Nrdp1 overexpression significantly decreased the number of colonies compared with controls, while even fewer colonies were formed following Nrdp1S overexpression (Fig. 5D and E). The EdU phenotype was the same as that observed for ErbB3 knockdown (Fig. 5F, G and H). These results indicate that Nrdp1S potentiates the effect of Nrdp1 suppression of ErbB3, thereby inhibiting glioma progression.

## Discussion

In this study, we showed that Nrdp1S, a novel short variant of Nrdp1, plays a synergistic role in the Nrdp1‐mediated ubiquitination and degradation of ErbB3 by stabilizing Nrdp1. Nrdp1S is produced by the insertion of 143 nucleotides into the 5′ region of Nrdp1, which results in the translation of an isoform with a shorter N‐terminus isoform through use of a downstream start codon. We speculate that this alternative codon usage reflects the DNA tertiary structure, folding state, regulatory sequence, or interactions with regulation factors.

Of the four Nrdp1 domains, the RING finger domain is responsible for TRIM protein interactions with E2 ubiquitin‐conjugating enzymes 23, 24, while the B‐box is a zinc‐binding domain with an unknown function 25. The coiled‐coil domain is involved in mediating protein–protein interactions, especially the formation of homotypic oligomers 26, 27. Finally, the carboxy‐terminal region is thought to be responsible for recruiting target proteins for ubiquitination at the E2 ubiquitin‐conjugating enzyme/E3 ubiquitin ligase complex 5, 16.

The C34S/H36Q (CHSQ) Nrdp1 mutant harbours two point mutations in its RING finger domain, which disrupt binding to E2 ubiquitin‐conjugating enzymes making it incapable of self‐ubiquitination *in vitro* 25. On the basis of this finding, we predicted that the truncated Nrdp1S would not be ubiquitinated or degraded. However, our results instead indicated that Nrdp1S can still be ubiquitinated and degraded in cells, despite lacking 71 aa at the N‐terminal. Further investigation showed that Nrdp1 and Nrdp1S can form homodimers as well as an Nrdp1–Nrdp1S heterodimer, suggesting that their fate depends upon which dimer is formed. Nrdp1–Nrdp1 homodimers will induce self‐ubiquitination and degradation; however, in the Nrdp1–Nrdp1S heterodimer formation, Nrdp1S might be ubiquitinated by Nrdp1, but cannot ubiquitinate Nrdp1 because of the lack of a RING finger domain. Nrdp1S–Nrdp1S homodimers are likely to be highly stable, because neither Nrdp1S can be ubiquitinated without a RING finger domain. Thus, as expected, our ubiquitination experiment showed that Nrdp1S undergoes less ubiquitination than Nrdp1. Taken together, we can conclude that Nrdp1S appears to function as a protector of Nrdp1 by attenuating its ubiquitination through the formation of an Nrdp1–Nrdp1S heterodimer.

Another short form of Nrdp1, Nrdp1 32, including only coiled‐coil and carboxy‐terminal domains (aa135–317), has been identified as a domain negative of Nrdp1 7, 16. Sequence comparison shows that Nrdp1S contains an additional domain (aa72–134) to Nrdp1 32, which is located in the B‐box domain. Our experimental evidence also indicates that Nrdp1S has an opposite role to Nrdp1 32 in regulating ErbB3 degradation. This might be of relevance in defining the function of the B‐box domain, which is currently unknown.

Nrdp1 has been reported to induce apoptosis and suppress tumour formation 27. Consistent with these reports, we demonstrate in the present study that overexpression of Nrdp1 promotes apoptosis and reduces cell tumourigenicity *in vitro*. Our results also show that Nrdp1S can stabilize Nrdp1 and more effectively inhibit tumour growth. Thus, it appears that Nrdp1S is also a tumour suppressor, and that Nrdp1S deficiency may contribute to the progression of glioma. However, the precise physiological function of Nrdp1S remains to be elucidated, including how its expression is regulated, and whether such expression is associated with particular human cancers.

## Conflicts of interest

The authors declare no competing financial interests.
